# The Effect of Chronic Immunosuppressive Regimens Treatment on Aortal Media Morphology and the Balance between Matrix Metalloproteinases (mmp-2 and mmp-9) and Their Inhibitors in the Abdominal Aorta of Rats

**DOI:** 10.3390/ijerph19116399

**Published:** 2022-05-24

**Authors:** Anna Surówka, Kamila Szumilas, Aleksandra Wilk, Kamila Misiakiewicz-Has, Kazimierz Ciechanowski, Karolina Kędzierska-Kapuza

**Affiliations:** 1 Department of Nephrology, Transplantology and Internal Medicine, Pomeranian Medical University, 70-111 Szczecin, Poland; anna.surowka@pum.edu.pl (A.S.); kazcie@pum.edu.pl (K.C.); karolina.kedzierska@gmail.com (K.K.-K.); 2 Department of Physiology, Pomeranian Medical University, 70-111 Szczecin, Poland; 3 Department of Histology and Embryology, Pomeranian Medical University, 70-111 Szczecin, Poland; aleksandra.wilk@pum.edu.pl (A.W.); kamila.misiakiewicz.has@pum.edu.pl (K.M.-H.); 4 Clinical Department of Gastroenterological Surgery and Transplantation, Postgraduate Medical Education Centre in Warsaw, 02-507 Warsaw, Poland

**Keywords:** immunosuppressive drugs, abdominal aorta, aortal media, metalloproteinases, metalloproteinase inhibitors, morphology, morphometry

## Abstract

Immunosuppressive drugs are widely and chronically used to avoid graft rejection in transplant recipients. However, they are also known to have organotoxic effects and can exert numerous side effects. The aim of this study was to assess whether the chronic treatment of rats with the most commonly used clinical immunosuppressive regimens in organ recipients had an effect on the morphology and function of the aorta. The rats were divided into five groups and each group was chronically treated with different sets of three immunosuppressive drugs (TRG, CRG, MRG, CMG, TMG) for 6 months. The changes were most profound in calcineurin inhibitor-based protocols. TMG protocol treatment was characterized by the most numerous alterations such as morphological changes, changes in the thickness of the tunic media, wider distances between elastic lamellae, an increase in the number of vSMCs and changes in collagen deposition. We concluded that the morphological changes were connected with MMP-2 and MMP-9/TIMP-2 and TIMP-1 imbalances, which was also determined and noticed.

## 1. Introduction

Currently the demand for organ transplants is increasing. Immunosuppressive therapy is necessary in all patients who receive organ transplants to suppress the immune response to the transplanted organ, effectively preventing rejection and allowing long-term survival of the allograft. In addition to the expected therapeutic effect, immunosuppressive drugs (IDs) exhibit serious side effects, such as toxic effects on various organs, e.g., the liver (hepatotoxicity) and kidneys (nephrotoxicity) [1,2].

Recently, an increased interest in the negative impact of chronic immunosuppressive therapy on the cardiovascular system, including patients after organ transplantation, has been observed [3]. The cause of organ loss in 50% of the above mentioned patients is chronic graft dysfunction or a supervening diagnosis of cardiovascular disease, neoplasm, or infection [4].

On the other hand, immunosuppressive treatment regimens play a vital role in transplantology. Their use by patients after organ transplants reduces the frequency of episodes of acute rejection and determines the survival time of the transplanted organ. The above-mentioned therapy is based on the use of combined agents from four main groups of drugs: (i) calcineurin inhibitors (CNIs) such as cyclosporine A (CsA) and tacrolimus (TAC); (ii) mTOR inhibitors (mTORi) (mammalian target of rapamycin (mTOR) inhibitors) including rapamycin; (iii) antimetabolites, drugs that inhibit cell division including mycophenolate mofetil (MMF); and (iv) glucocorticosteroids (GCS), including prednisone [5,6].

These drugs suppress components of the humoral and cellular immune response. As a result, the levels of cytokines are decreased. The signal transmission between components of the immune system is disturbed and the proliferation of the immune system cells is reduced [7].

The aorta is one of the largest arteries in the human body and is classified as a large and elastic artery. A key component of aorta wall is extracellular matrix (ECM), and its composition and spatial organization is essential for the unique physiological function of the aorta. The aortic wall is built with three layers: the tunica intima, tunica media and tunica adventitia [8].

The tunica intima, the innermost layer of the aortic wall, is lined with a simple squamous epithelium—the endothelium resting on the basement membrane. The basement membrane of the endothelium contains ECM elements typical for these structures, such as laminin, type IV collagen, fibronectin, perlecan and glycosaminoglycan—heparan sulphate, enabling the regulation of processes such as the proliferation, migration and survival of the endothelial cells [9,10]. The tunica intima has an internal elastic lamina adjacent to the tunica media, the next layer of the aortic wall. The media consists of multiple layers of vascular smooth muscle cells (vSMC) separated by numerous fenestrated elastic lamellae. Owing to its concentric elastic lamellae and vSMCs, this layer is rich in ECM elements including glycosaminoglycans (GAGs), proteoglycans and glycoproteins, as well as collagen types I and III [11]. The vSMCs and elastic fibers in the media form elastin-contractile units, while the fibrillar collagen are responsible for the tensile strength [12]. Two different vascular smooth muscle cell populations can be identified by their spindle or epithelioid/rhomboid shape and their differentiated versus proliferative phenotypes [13,14].

The outer elastic lamina is the boundary between the tunica media and tunica adventitia, the outermost layer of the aortic wall, formed by connective tissue, rich in fibroblasts, collagen and elastic fibers. It is innervated and contains vasa vasorum and lymphatic vessels [8].

The extracellular matrix, depending on its location, is produced by vSMCs (media) and fibroblasts (adventitia). The composition of the ECM and its integrity in the aortic wall determine its mechanical properties. The extracellular matrix of the vessels undergoes constant physiological reconstruction and degraded proteins are synthesized de novo [8].

Disturbances in the ECM remodeling process may lead to the development of pathological vascular changes such as atherosclerosis, aneurysms and chronic venous insufficiency, and others [15,16,17,18,19,20]. Vascular integrity and homeostasis is maintained by the dynamics with which the ECM interacts with cellular components. The structure and interactions of the ECM are controlled by enzymes from the family of metalloproteinases (MMPs) [21,22].

Under physiological conditions, endothelial and smooth muscle cells control the secretion of MMPs and their inhibitors (TIMPs—metalloproteinase inhibitors) at a constant level. This allows maintaining a balance between the degradation and synthesis of the elements in the blood vessel wall. Due to their substrate specificity, metalloproteinases are divided into five main groups. Two of them, MMP-2 and MMP-9, are widely recognized markers for remodeling the artery wall [23,24]. They belong to gelatinases, which are characterized by the ability to hydrolyze proteins such as collagen type III, collagen type IV, collagen type V, elastin, laminin, fibronectin [25,26,27]. The inhibitors of MMP-2 and MMP-9 are TIMP-2 and TIMP-1, respectively. Under the influence of stress factors, and consequently as a result of a response to inflammation, the expression of MMPs is increased [17,28,29,30,31]. Moreover, Blankenberg et al. (2003) and Ferroni et al. (2003) reported a positive correlation between the increased concentration of MMP-9 in blood serum and the occurrence of acute coronary syndrome in patients. Thus, the control of expression and the activity of enzymes that degrade ECM proteins play a key role in the proper structure and function of blood vessels [32,33]. A disturbed balance between the activity of MMPs and TIMPs plays a fundamental role in the pathological remodeling of extracellular components and in cell migration and proliferation, ultimately leading to dysfunctional changes in the blood vessels. A correlation has been observed between the increased activity of gelatinases and diseases such as coronary artery disease, aneurysms, peripheral arteriosclerosis, and arterial hypertension [3].

Currently, there is an insufficient number of studies in the literature showing the effect of individual treatment regimens, with triplet of immunosuppressive drugs, on morphological changes in vessels or the expression of MMP-2, MMP-9 and TIMP-1, TIMP-2. Obtaining such knowledge seems to be extremely important due to the possibility of eliminating some risk factors, such as aortic wall dissection, as an example of chronic corticosteroid use [34]. Therefore, the aim of this study was to determine whether chronic treatment of rats with the most commonly used clinical immunosuppressive regimens in organ recipients had an effect on the aortic morphology and function of the animals.

## 2. Materials and Methods

### 2.1. Research Material

**Figure 1:**
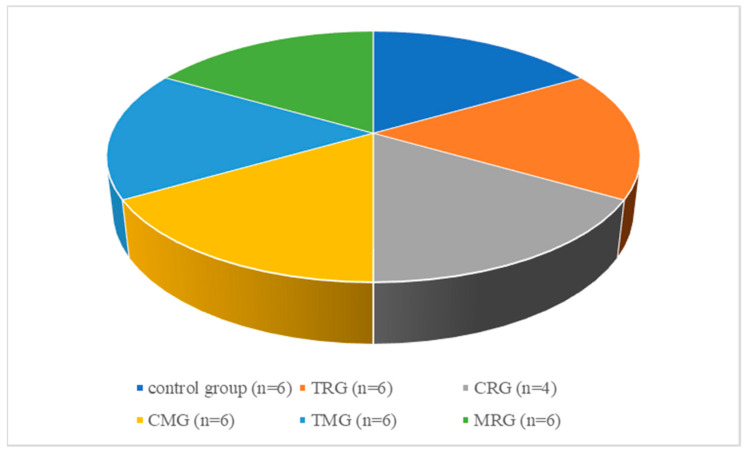
Drug protocols used in the current experiment.

The biological material of the current study was rat aortas that were the archival material accumulated in the Department of Nephrology, Transplantology and Internal Medicine, Pomeranian Medical University in Szczecin, obtained from an experiment performed previously by Kędzierska et al. (2013). This study was approved by the Szczecin Local Ethical Committee for Experiments on Animals (decision no. 24/2008, dated 24 November 2008) [35]. The animals were divided into six groups: the control group (C) and 5 experimental groups—six rats/group, however, two rats from the CMG group died during the experiment. The animals of the control group did not receive any drugs, while the rats of the individual experimental groups were administered different combinations of immunosuppressants, according to the most commonly used treatment models in kidney recipients: tacrolimus (T); cyclosporin A (C); rapamycin (R); mycophenolate mofetil (M); glucocorticosteroids (G) (Figure 1). The following doses of drugs were used in the experiment: tacrolimus (Prograf)-4 mg/kg/day, mycophenolate mofetil (Cellcept)-20 mg/kg/day, cyclosporin A (Sandimmun-Neoral)-5 mg/kg/day, rapamycin (Rapamune)-0.5 mg/kg/day, prednisone (Encorton)-4 mg/kg/day. The mean body weight of the rats was 305 g. The rats gained weight during the experiment, so the dose of each drug was changed after 3 months of the experiment, adjusting for the body weight of the animals. After 6 months of the experiment, the mean body weight of the rats in each group was as follows: control 573.2 g; TRG 403 g; CRG 467 g; MRG 435.6 g; CMG 544 g; TMG 482.5 g. There was a statistically significant difference between the control group and the TRG group. In the experiment the animals were treated with the pharmaceutical form of each used drug for 6 months in accordance with formerly published data [35].

### 2.2. Morphological Studies

The aorta samples were fixed in freshly prepared 4% paraformaldehyde and embedded in paraffin, using routine procedures. For the morphological analysis, serial slices (3–4 μm thickness) of the aorta of the control and experimental rats were mounted on poly-L-lysine coated glass slides and stained with hematoxylin and eosin (H-E). Elastic fibers in wall of aorta were identified with orcein (from purplish red to brown) (Bio-Optica Milano, Italy). To visualize collagen fibers, the slides were stained with Mallory trichrome (Bio-Optica Milano, Italy) and for an additional 30 min with Picro Sirius Red (Direct Red 80 Sigma Aldrich—0.1% of Sirius Red in saturated aqueous picric acid), as described by Junqueira et al. [36]. Silver impregnation was performed to visualize reticular fibers (Bio-Optica Milano, Italy). All histochemical reactions were carried out according to the manufacturer’s protocols.

### 2.3. Morphometric Studies

The sections of aorta stained with H-E were used for morphometric analyses. This part of the analysis was performed using Case Viewer, digital microscopy application [37]. All analyses were performed on slides under 40× objective magnification. The thickness of the tunica media (as there is a clear border, the measurement was carried out with the tunica intima) and the distance between elastic lamellae of aorta were analyzed for each rat of each group, except CRG (*n* = 4). These parameters were expressed in µm. Within each group, 10 measurements for each rat were performed. The number of vascular smooth muscle cell (vSMC) nuclei in the tunica media was counted using a rectangular mesh (45.4 μm × 60.5 μm).

### 2.4. The Morphoquantitative Assessment of the Area Occupied by Collagen Fibers in the Aortal Media

The content of collagen fibers in the tunica media in the aorta of the rats were quantified with the use of cross-platform software ImageJ in slides stained with the Mallory Trichrome method, using a microscope objective with ×40 magnification. Four random areas were selected for measurement from the aorta of each rat of each group. The area occupied by collagen fibers was expressed as a percentage.

### 2.5. Immunohistochemistry (IHC)

To identify MMP-2 and MMP-9, and TIMP-2 and TIMP-1 expression, the following primary antibodies were used: mouse anti-MMP-2 at 1:250 (sc-53630; Santa Cruz Biotechnology, Inc., Santa Cruz, CA, USA), mouse anti-TIMP-2 at 1:250 (sc-21735; Santa Cruz Biotechnology, Inc., Santa Cruz, CA, USA); mouse anti-MMP-9 at 1:250 (sc-13520; Santa Cruz Biotechnology, Inc., Santa Cruz, CA, USA); mouse anti-TIMP-1 at 1:250 (sc-21734); Santa Cruz Biotechnology, Inc., Santa Cruz, CA, USA). All antibodies were diluted by Diluent (Agilent Dako EnVison, Hovedstaden, Denmark). All the antibodies used are recommended for the detection of MMP-2, MMP-9, TIMP-1 and TIMP-2 of murine, rat and human origin by WB, IF and IHC (P).

Slides were deparaffinized in three changes of xylene and rehydrated in a graded series of ethanol to distilled water. For antigen retrieval, slides were placed in 0.01 M citrate-buffer pH 6.0 and heated in a microwave for 10 min. Endogenous peroxidases were quenched by incubation in a Dual Endogenous Enzyme Block (Agilent Dako EnVison, Denmark) for 10 min. Next, sections were incubated for 30 min at room temperature with the primary antibodies mentioned above. Subsequently, sections were incubated with labeled polymer (Labeled Polymer HRP) (Agilent Dako EnVison, Denmark) (30 min), and then a 10-min incubation was performed with a substrate–chromogen complex with 3,3′-diaminobenzidine (DAB +) (Agilent Dako EnVison, Denmark), causing a brown precipitate to form at the site of the antigen. Negative controls were made by eliminating the primary antibody. After counterstaining with hematoxylin (Sigma-Aldrich), specimens were mounted in the permanent mounting medium. Immediately prior to each incubation, tissues were washed twice in PBS for 5 min and placed in a TBS bath for 5 min. Each incubation took place in a humid chamber at room temperature [6]. Staining was performed according to the manufacturer’s protocol. After completion of the reaction, the slides were examined under a microscope (Leica DM5000B, Wetzlar, Germany).

#### Semi-Quantitative Assessment of MMP-2, MMP-9, TIMP-1 and TIMP-2 Expression

The immunoexpression of MMP-2, MMP-9, TIMP-1 and TIMP-2 in the tunica media in the aorta of rats were quantified in slides with the use of ImageJ Fiji software, according to the Crowe and Yue protocol [38]. Four areas of tunica media were selected from the aorta of rats in each group, using objective with magnification x40. The area occupied by products of IHC reactions was expressed as a percentage.

### 2.6. Statistical Analysis

Statistica 13 software was used for morphometry and the quantified analysis of the area of collagen fibers and IHC products in the tunica media, and results were expressed as arithmetic means ± standard deviation, medians and minimum and maximum values. The data were tested for normality with the use of the Shapiro–Wilk test. Since the data were non-normally distributed the Kruskal–Wallis test was used, followed by Dunn’s post hoc test for comparisons between groups. In all cases, *p* < 0.05 was considered statistically significant.

## 3. Results

### 3.1. Morphological Characters of Rat Aortas

**Figure 2:**
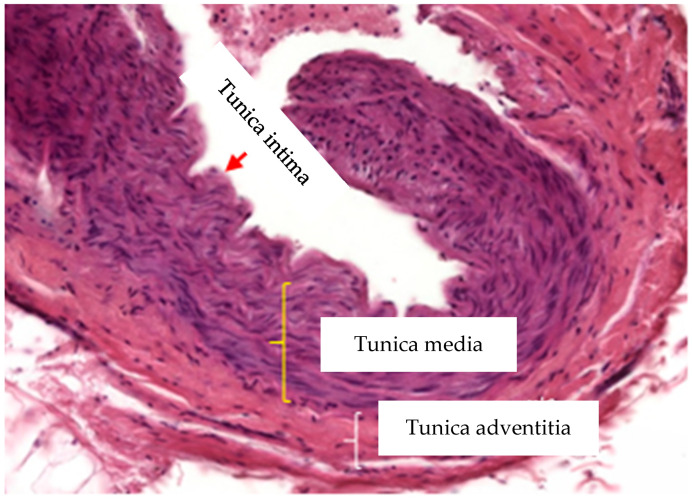
Tunics in wall of elastic artery.

**Figure 3:**
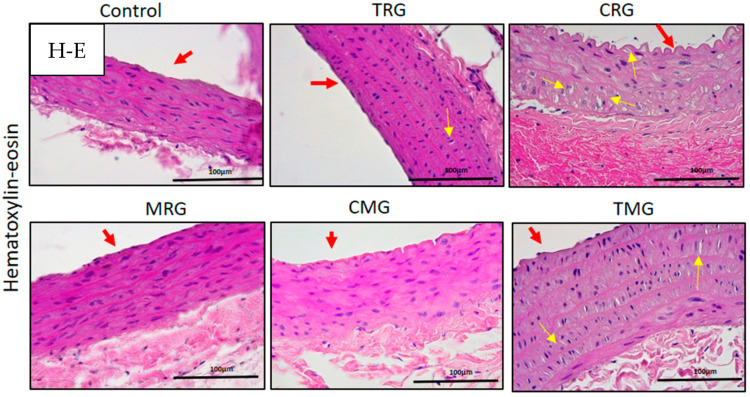
Aorta of rats treated with panels of immunosuppressive drugs. TRG, CRG, TMG—yellow arrows indicate changed shape of vascular smooth muscle cells in media; CRG—folded tunica intima. Red arrows indicate aortal intima. Hematoxylin-eosin (H-E) staining. Objective magn. ×40; bar 100 μm.

Representative histological longitudinal sections of aorta by H-E staining are shown in Figure 2. The wall of the control aorta presented a typical structure of tunics. Tunica intima consisted of a lining of endothelial cells and thin underlying layer without clear limitation from the tunica media. The tunica media contained a regular arrangement of elastic lamellae, and the nuclei of vascular smooth muscle cells between them were visible. Tunica adventitia, the outermost part, contained elements of connective tissue. There were no abnormalities visible in the wall of the aorta in rats treated with MRG and CMG. Changes in shape from longitudinal to oval SMC nuclei in the media of rats treated with TRG were observed. The most pronounced alteration was expressed in the media of rats treated with CRG and TMG immunosuppressants. A round shape and hyperplasia of the vascular smooth muscle cells in the tunica media were visible. Moreover, the folding of endothelium of tunica intima was found in CRG rats (Figure 3, arrows).

**Figure 4:**
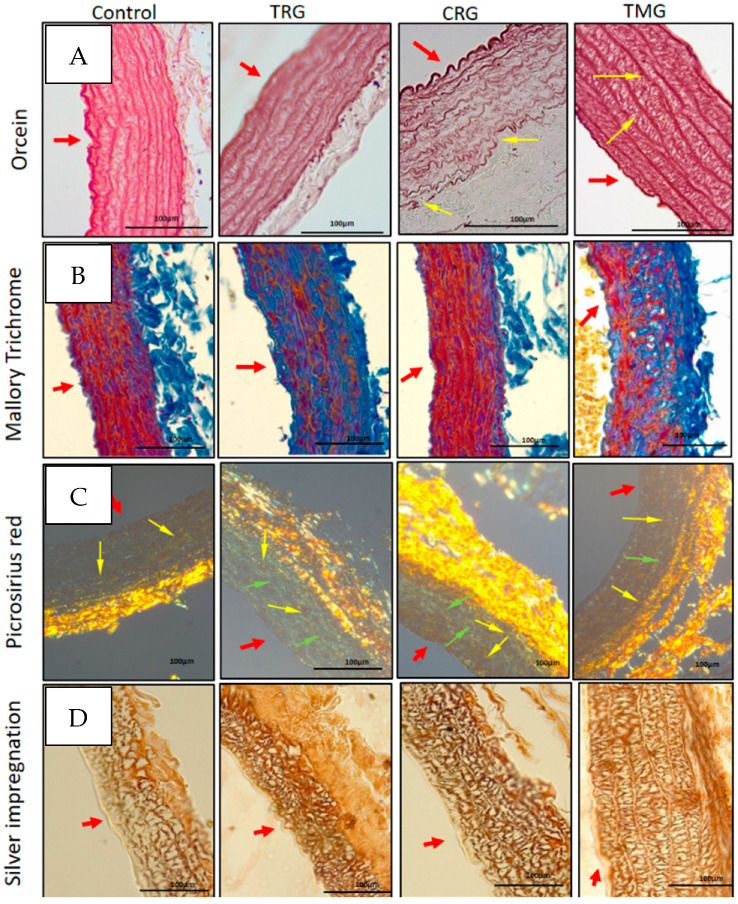
Visualization of elastic (**A**) and collagen fibers (**B**–**D**). Red arrows indicate aortal intima. (**A**): yellow arrows indicate fragmentation of elastic fibers in lamellae in CRG and thickening of elastic lamellae in media in TRG groups; (**B**)—collagen fibers accumulation in media (deep blue); (**C**)—collagen type I with yellow color and strong birefringence (yellow arrows) and collagen type III with greenish color and weak birefringence (green arrows); (**D**)—type III collagen in aortal media (**D**). Orcein staining (**A**); Mallory trichrome staining (**B**); Picro-Sirius Red—Polarizing microscopy(**C**); Silver impregnation (**D**). Objective magn. ×40; bar 100 μm.

Staining with orcein showed the arrangement of the elastic lamellae in the tunica media of the control and experimental groups. A thickening of the lamellae (arrows) was visible in the media of TMG rats and additionally, a disintegration of the arrangement and local fragmentation of elastic fibers (arrows) in the tunica media of rats treated with CRG drugs was observed (Figure 4A).

In Mallory Trichome method of staining, collagen fibrils are deep blue, the nuclei of cells are red, and elastic fibrils are yellow. The collagen fiber content was increased in the tunica media of the aorta in rats treated with TRG, CRG and TMG immunosuppressants (Figure 4B).

Aorta sections stained with Picro Sirius red were analyzed by polarized microscopy (Leica DM5000B, Wetzlar, Germany) to detect the color and intensity of birefringence of collagen fibers in the media of each group of animals. The thin collagen fibers that displayed a greenish color and weak birefringence were considered collagen type III. The larger collagen fibers showed a yellow or yellowish-orange color and a strong birefringence, these were considered collagen type I. The tunica media of control rats mainly showed the presence of the greenish color type III collagen (green arrows) and a moderate content of type I collagen displaying a yellowish color (yellow arrows). An increased content of type III collagen (green arrows) was observed in the media of rats treated with TRG, and an increased content of collagen type I with yellow birefringence was visible in the aorta of the TMG rat group (Figure 4C).

The visualization of type III collagen revealed a higher content of this collagen type in the media of the aorta in rats treated with TRG and CRG and moderate in the media of TMG rats (Figure 4D).

### 3.2. Morphometric Analysis

The morphometry regarding the thickness of the tested aortas indicated that the thickest tunica media was present in the TMG group, whereas the thinnest was found in the CRG group. Significant differences were found between the control group and every other treatment group. Additionally, parameters were also statistically significant between TRG vs. CRG and TRG vs. CMG. The largest distance between the elastic lamellae of the tested aortas was observed in the TMG group and it was over 50% larger than in the CMG group, which has the shortest distance between lamellae. Moreover, a significantly smaller length of the above-mentioned parameter was shown in the control group when compared with the MRG and TMG group. Additionally, a significant difference was confirmed between the TRG and TMG group. The greatest number of nuclei of vSMCs (number of nuclei in morphometry) was observed in the TMG group and it was almost three times more than in the MRG group. Interestingly, significant differences regarding the above-mentioned parameter was indicated between control and all the treatment groups, except the CRG group. The number of nuclei were significantly different in every treatment group (Table 1).

### 3.3. Assessment of the Area of Collagen Fibers in Aortal Media

The percentage of the area occupied by collagen fibers was evaluated in the tunica media of the aorta in control and experimental rats. A statistical comparison of the treatment groups showed that groups with tacrolimus-based protocol (TRG, TMG) had the largest area with collagen fibers when compared with the control and Rapa-, CsA-, and MMF-based protocol groups (Table 2), and each triple-drug immunosuppression group (Table 3).

### 3.4. Immunohistochemistry

**Figure 5:**
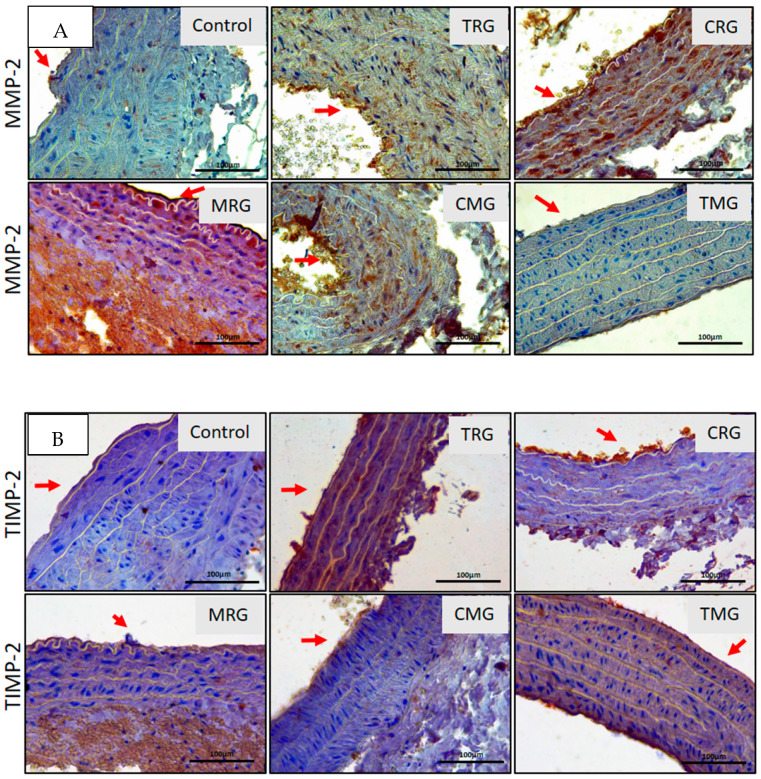
Immunolocalization of MMP-2 (**A**) and TIMP2 (**B**) in aorta of rats in all groups. Red arrows indicate aortal intima. IHC A and B. Objective magn. ×40; bar 100 μm.

Since the changes in the content of collagen depositions indicated alterations in its turnover, we decided to study the immunoexpression of two matrix metalloproteinases MMP-2 and MMP-9 (MMPs), and their inhibitors—TIMP-2 and TIMP-1—major regulators of collagen turnover, in the wall of the aorta in control and experimental rats. The expression of MMP-2 was observed in the tunica media extracellular matrix (ECM) of TRG, CRG, MRG and CMG rats. No expression of MMP-2 was observed in the media of control and TMG rats. The most pronounced expression was noted in the media ECM of CRG and MRG rats (Figure 5A). Except for the TMG group, MMP-9 expression was not affected by immunosuppressive drug panels (Figure 5A).

**Figure 6:**
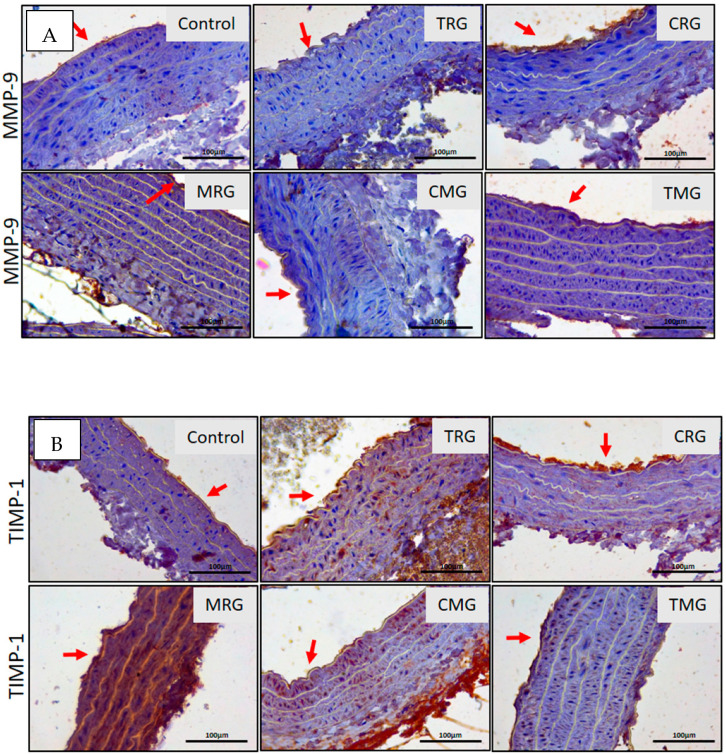
Immunolocalization of MMP-9 (**A**) and TIMP1 (**B**) in wall of aorta in all groups of animal. Red arrows indicate aortal intima. IHC A and B. Objective magn. ×40; bar 100 μm.

The expression of inhibitors of the tested MMPs was also assessed. The main inhibitor of MMP-2 is TIMP-2, while TIMP-1 inhibits activity of MMP-9. The expression of TIMP-2 (Figure 5B) was only identified in the tunica media of TRG rats, while expression of TIMP-1 (Figure 6B) was noted in the tunica media of TRG, CRG, MRG and CMG rats (Figure 5B). TIMP-1 expression was not found in the aortal media of Control and TMG rats (Figure 6B).

We have divided the analyses into two parts: the comparison of (i) the area of immunoexpression of MMPs and their inhibitors in control groups versus five experimental groups; (ii) the balance between MMPs and their inhibitors. The results regarding expression of MMPs and their TIMPs suggest that MMP-2 significantly increased in CRG compared to the control group and the percentage area of MMP-2 was almost five times more expansive. Interestingly, the area of expression of MMP-2 in the MRG group significantly decreased in comparison to the untreated group. A comparison of the area of expression of their inhibitor (TIMP-2), showed that TRG, MRG and CMG groups exhibited significant changes vs. the control group. Regarding TIMP-1, significant alterations in TRG, MRG, TMG were also observed vs. control. Our results showed that control, TRG, CRG and CMG groups indicated an increased MMP-2 expression comparing to its inhibitor. A comparison of the MMP-2/TIMP-2 balance indicated an increase in the area with immunoexpression of TIMP-2 in only two groups, MRG and TMG.

A comparison of treated groups with control groups suggests that the expression of MMP-9 significantly increased in the TMG group. The area of significantly altered TIMP-1 vs. control is noticeable in TRG, MRG and TMG groups. In respect of the balance between MMP-9 and TIMP-1, three groups indicated increased TIMP-1 expression vs. MMP-9 expression (TRG, CRG, MRG). Additionally, the TMG group had a higher expression of MMP-9 vs. TIMP-1. Both areas of expressions of enzymes and their inhibitors were visible at the same degree in the control group and CMG group.

Moreover, area with immunoexpression of the MMPs and their TIMPs indicated significant alterations between treated groups (Table 4).

## 4. Discussion

It is known that vSMCs play an important role in the remodeling of the ECM of media wall and can be involved in pathological processes [39]. Therefore, disorganization of ECM elements can reduce aortic elasticity and can affect overall vessel mechanics [40,41].

The data obtained in the present study allow us to establish that the chronic treatment of rats with the most common clinical immunosuppressive regimens may cause disturbances in the morphology and function of the abdominal aorta. In routine stained slides, we observed some abnormalities such as the loosening of medial tissue, wider distances between elastic lamellae, degradation of elastic fibers, degeneration and changes in the shape of smooth muscle cell nuclei, hypertrophy and their hyperplasia.

There were no clearly visible morphological changes in the aortal media of rats treated with an MRG and CMG drug combination, and no significant differences in the thickness of the media were observed in either group of animals. Perhaps the lack of clear changes in morphology were associated with the protective role of rapamycin since it regulates the metabolism, proliferation, migration and survival of aortal cells [42]. Moreover, it was shown that, after the vascular injury, rapamycin can inhibit the proliferation and migration of smooth muscle cells [43]. A study by Zhou et al. (2019) on a mouse model with induced thoracic aortic aneurysm and dissection (TAAD) showed that rapamycin significantly reduced TAAD formation, elastic fiber destruction, and inhibited MMP-9 and MMP-2 synthesis to maintain the structure of the aortic wall. The treatment of mice with rapamycin caused dramatically reduced inflammatory cell infiltration in the aortic wall [44]. The protective effect of rapamycin was also confirmed in another in vivo (mice) and in vitro (SMCs cultures) study performed by Hayashi-Hori et al. (2020), by using agents which stimulate the development of changes characteristic for aortal dissection (AD). It was shown that rapamycin exerted the beneficial effect associated with the inhibition of SMC proliferation through selectively suppressing the cell cycle-related gene subnetwork and maintained the contractile not proliferative phenotype of SMCs in the aortal media [45].

In this study, the most pronounced changes in morphology were observed in the aortal tunica media of rats receiving triple-drug immunosuppression such as TRG, CRG and TMG. The morphological changes were connected with an abnormality in the aortal smooth muscle cells; as they degenerated, the nuclei in control was spindle-shaped whereas in the experimental groups, the nuclei was epitheliod/rhomboid shaped, indicating a changing phenotype of the cells. The shape of vSMCs depends on the organization of ECM components, and the mechanical strain regulates phenotype, function and matrix remodeling. Under the physiological conditions, vSMCs that are spindle in shape present a contractile phenotype [13,46].

Regarding the thickness of the wall, statistically significant differences in the thickness of the media between the control and all experimental groups were observed. The widest thickness of the middle layer of the aortic wall was observed in the TMG group compared to the control group whereas the CRG group was thinnest. Regarding calcineurin inhibitors (CNIs), tacrolimus is known as a much more toxic drug when compared to cyclosporine A. The next examined parameters were also altered compared to the control. The distances between elastic lamellae were statistically higher in the MRG and TMG groups; moreover, there were statistical differences in the numbers of vSMCs between the control and the TRG, MRG, CMG, TMG groups.

The changes could be indicative of the harmful effects of CNIs on aortal morphology, which can be caused by a lower dose of the inhibitors. In a study by Koskinen et al. (1995), rats with cardiac allograft were treated with methylprednisolone, azathioprine, and three different doses of cyclosporin A. After three months of treatment, cyclosporine in 10 mg/kg/day significantly inhibited intimal thickening in arteries; however, the highest dose of cyclosporine A reduced the thickness [47]. Similar results were obtained in an experiment by Soukiasian et al. (2004) with rats who had a cardiac transplant. They found that treatment with cyclosporine inhibited arteriosclerotic vascular changes in the allograft [48]. Tacrolimus, the other CNI, was also included in triple-drug immunosuppression in the experiment. Both cyclosporine and tacrolimus are able to reduce the rate of allograft rejection; however, their chronic use can be characterized by a number of side effects, including vascular toxicity [49]. It was earlier hypothesized that transplant vasculopathy (TV) represented an ineffective delayed-type-hypersensitivity response directed against donor endothelial cells and SMCs in the media [50]. Furthermore, it was indicated that calcineurin inhibitors can increase proinflammatory cytokine production and markers of endothelial activation and SMCs, both in in vitro and ex vivo [49]. Under these conditions, vSMCs can be stimulated to proliferation and migration [51]. In our experiment we did not measure the level or immunoexpression of proinflammatory cytokines; however, it was noted that the mean number of vSMC nuclei had been two and a half times higher in TMG than in the control group.

Immunosuppressive drugs with mycophenolate mofetil, an antiproliferative agent, together with corticosteroid can reduce the fibrosis process and prevent intimal hyperplasia [52]. In our experiment, the media of the aorta was altered in the group of rats treated with TMG immunosuppressants. It could be speculated that the protective effects of mycophenolate mofetil and corticosteroid were reduced by tacrolimus. In our study, an increased accumulation of collagen fibers in the aortal media had been observed in rats treated with Tac-based protocols (TRG, TMG—89.4% vs. Control 45.6% and other groups). This process could be connected with the activity of metalloproteinases and the expression of their inhibitors during the treatment of rats with immunosuppressive drugs.

According to Bianchi et al., an increased expression of MMP-2 in Control, TRG, CRG and CMG compared with the expression of TIMP-2 may suggest that the increase in the aforementioned metalloproteinase is a form of compensatory mechanism against the excessive accumulation of collagen resulting from stressful conditions such as CsA therapy. In a previous study, they also showed that CsA caused intense myocardial fibrosis and the increase in MMP-2, while MMP-1 and MMP-9 levels were unchanged. Our results correlate with their data, since tacrolimus belongs to CNIs and exhibit similar pharmacological properties to CsA [53].

In our study, the MRG and TMG groups exhibited increased TIMP-2 expression as compared to MMP-2. Waller et al. (2005) showed that the use of CNIs (cyclosporine, tacrolimus) or rapamycin in combination with MMF reduced the expression of collagen III, inhibited intimal thickening and decreased the expression of MMP-2, MMP-9 and TIMP-1 compared to monotherapy with the drugs mentioned. The best results were obtained when MMF was combined with rapamycin. It was concluded that MMF reduces the expression of factors promoting the proliferation and migration of vascular smooth muscle cells and inhibits intimal hypertrophy. In our study, the MRG group did not increase the expression of MMP-9; moreover, the area of expression of MMP-2 significantly decreased compared to the untreated group [52].

In the rapamycin-based protocols group (TRG, CRG and MRG), the expression of TIMP-1 increased compared to the expression of MMP-9. There are multiple studies proving the antifibrotic effects of rapamycin, which has also been confirmed by us. Increased activity of TIMPs can reflect the inhibition of fibrosis because the activity of matrix degrading enzymes is inhibited. The similar results were obtained by Osman et al. (2011) in studies regarding mTOR inhibitor—rapamycin and its effect on MMP-9/TIMP-1 balance in in vitro conditions [54]. Brook et al. (2005) found that a suitable combination of lower doses of cyclosporin (7.5 mg/kg/d) + rapamycin (0.5 mg/kg/d) administered to the rat model gives an antiproliferative/antifibrotic effect through an increase in MMP-2 expression and a decrease in TIMP-1 expression [55]. Our results suggest that MMP-2 expression significantly increased in CRG compared to the control group and all treatment groups.

There were no clearly visible morphological changes and no increase of area percent of collagen fibers in the aortal media of rats treated with this drug combination. The thickness of the tunic in this group was also lower compared to that of the control.

Regarding the MMP-9/TIMP-1 balance, only the TMG group exhibited increased MMP-9 versus its inhibitor, which leads to the reorganization of ECM. Perhaps Tac in combination with MMF exhibits more profibrotic properties, since it is commonly known that Tac is much stronger and more toxic than another calcineurin inhibitor—CsA [56].

Moreover, the comparison of the area of collagen fibers in tunica media of aorta due to the main drug-based protocols showed a significant increase in all treatment groups vs. the control group. Additionally, the highest percentage of area with collagen fibers (almost 90%) in Tac-based protocols was observed. Similar results were obtained by Khanna et al. [57]. Biopsies from patients with a histological diagnosis of CsA or Tac nephrotoxicity demonstrated that tacrolimus treatment significantly increased the expression of collagen by about 300% in the patients with tacrolimus nephrotoxicity compared to patients with CsA nephrotoxicity [55]. In another study, Waller et al. (2004) presented the effect of one drug: cyclosporine, tacrolimus or rapamycin on the development of intimal thickening in the rats model. In their study, treatment with each immunosuppressive drug resulted in increased ECM deposition. The tested drugs significantly inhibited the expression of MMP-2, MMP-9 and TIMP-1 genes, but CsA increased the expression of both MMPs compared to the expression of TIMP-1. Perhaps, the difference in the expression of MMPs versus TIMP is the cause of increased collagen deposition using Tac but not CsA, although both drugs are calcineurin inhibitors. In the above-mentioned study, Tac and CsA exhibited more expressed alterations in terms of allograft vasculopathy compared to rapamycin [58].

## 5. Conclusions

Our study indicates that immunosuppressive protocols affect the MMP/TIMP balance in the abdominal aorta which in consequence alter the morphology and function of the vessel. The changes in the CNI-based protocol group were the most profound.

### Strengths and Limitations of the Study

The strength of the study is undoubtedly the experimental design. The animals were treated with an immunosuppressive drugs protocol for six months, which reflects about 15 years of human life. Furthermore, immunosuppressive drugs were used in the experiment according to the treatment of organ recipients; therefore, we have observed the effect of the composition of three drugs, not one.

On the other hand, the limitation of the study is that the experiment was performed on animal tissue and only partly reflects the effects of immunosuppressive drug regimens on human tissue.

## Figures and Tables

**Table 1:** The parameters of morphometry of the aorta of rat treated with different immunosuppressive drugs protocols.

	Control (*n* = 6)	TRG (*n* = 6)	CRG (*n* = 4)	MRG (*n* = 6)	CMG (*n* = 6)	TMG (*n* = 6)
Width of *Tunica Media* (µm)						
AM ± SD	65.4 ± 3.09	79.83 ± 2.95	49.81 ± 3.34	75.71 ± 5.34	88.06 ± 2.87	123.81 ± 6.05
Median	65.3	79.75 *	50.3 *^,T^	76.0 *	88.15 *^,T^	122.3 *
Min–max	61.6–70	74.3–84.0	44.7–53.6	69.7–88.1	83.5–93.0	117.1–133.6
Distance between elastic lamellae in *Tunica Media* (µm)						
AM ± SD	9.26 ± 2.38	10.48 ± 2.82	9.62 ± 3.11	18.54 ± 24.41	10.2 ± 1.98	19.52 ± 5.41
Median	9.75	10.65	8.65	14.6 *	9.7	17.45 *^,T^
Min–max	3.3–14.6	4.8–14.8	2.6–19.1	5.9–143.0	6.6–13.7	11.5–39.0
Number of vascular smooth muscle cells nuclei in *Tunica Media*						
AM ± SD	53.83 ± 4.53	72.33 ± 3.14	54 ± 7.48	39.66 ± 6.12	99.50 ± 10.05	138.66 ± 8.14
Median	55.5	72.5 *	55.0 ^T^	39.0 *^,T,C^	98.50 *^,T,C,M^	139.0 *^,T,C,M^
Min–max	46.0–58.0	68.0–77.0	44.0–62.0	32.0–50.0	89.0–111.0	128.0–151.0

AM: arithmetic mean; SD: standard deviation; *n*—number of measured parameters, Min–Max—minimum and maximum values; * *p* < 0.05 vs. control; ^T^ *p* < 0.05 vs. TRG; ^C^ *p* < 0.05 vs. CRG; ^M^ *p* < 0.05 vs. MRG.

**Table 2:** Area of collagen fibers in tunica media of aorta in the study groups of rat.

Parameter/Group	Control (*n* = 24)	TRG (*n* = 24)	CRG (*n* = 16)	MRG (*n* = 24)	CMG (*n* = 24)	TMG (*n* = 24)
Area of collagen fibers in tunica media of aorta (%)						
Mean ± SD	43.3 ± 19.6	85.25 ± 4.8	70.0 ± 4.06	57.89 ± 11.81	66.81 ± 8.21	92.36 ± 6.21
Median	45.56	86.24 *	70.0 *	58.3 ^T^	66.46 ^T^	94.02 *^,C,M,∆^
Min–Max	10.62–74.65	73.3–92.21	61.84–5.32	27.07–73.97	55.58–85.46	76.06–97.97

AM: arithmetic mean; SD: standard deviation; *n*—number of measured areas; Min–Max—minimum and maximum values * *p* < 0.05 vs. control; ^T^ *p* < 0.05 vs. TRG; ^C^ *p* < 0.05 vs. CRG; ^M^ *p* < 0.05 vs. MRG; ^∆^ *p* < 0.05 vs. MRG.

**Table 3:** Area of collagen fibers in tunica media of aorta due to main drug-based protocols.

Parameter/Group	Control (*n* = 24)	Tac—Based Protocols (TRG, TMG) (*n* = 48)	CsA—Based Protocols (CRG, CMG) (*n* = 40)	MMF—Based Protocols (MRG, CMG, TMG) (*n* = 72)	Rapa—Based Protocols (TRG, CRG, MRG) (*n* = 64)
Area of collagen fibers in tunica media of aorta (%)					
Mean ± SD	43.31 ± 19.6	88.81 ± 6.56	68.08 ± 7.0	72.3 ± 17.2	71.18 ± 14.37
Median	45.56	89.35 *	67.28 *^,T^	68.18 *^,T^	71.75 *^,T^
Min–Max	10.62–74.61	73.3–97.97	55.58–85.46	27.07–97.97	26.47–92.21

AM: arithmetic mean; SD—standard deviation; *n*—number of measured areas; Min–Max—minimum and maximum values * *p* < 0.05 vs. control; ^T^ *p* < 0.05 vs. Tac—based protocol.

**Table 4:** The expression MMP-2 and MMP-9, and their inhibitors—TIMP-2 and TIMP-1 in tested groups. Relations between MMPs expression and their inhibitors in relation to maintaining equilibrium or imbalance.

Parameter/Group		Control (*n* = 24)	TRG	CRG	MRG	CMG (*n* = 24)	TMG
			(*n* = 24)	(*n* = 16)	(*n* = 24)		(*n* = 24)
Area of MMP2 in tunica media of aorta (%)	Mean ± SD	11.24 ± 4.64	8.3 ± 3.57	47.16 ± 21.04	3.29 ± 1.87	10.39 ± 6.42	7.94 ± 2.42
	Median	9.7 ^C^^,M^	8.83 ^C,M^	51.78 *^,T,M,∆,^^	3.21 *^,T,C,∆,^^	9.88 ^C,M^	7.48 ^C,M^
	Min–Max	5.79–21.34	1.75–4.11	8.73–79.98	0.84–8.01	1.05–25,32	4.42–15.02
Area of TIMP2 in tunica media of aorta (%)	Mean ± SD	2.18 ± 1.09	7.19 ± 4.4	6.71 ± 5.84	13.01 ± 9.08	1.53 ± 1.02	11.75 ± 9.48
	Median	2.18 ^T,M,∆^	6.83 *^,T,C,∆,^^	3.71 ^M,∆^	11.72 *^,C,^^	1.34 *^,T,C^	11.03 ^T,M^
	Min–Max	4.4–16.19	0.00–0.33	2.22–1.84	0.02–0.61	0.12–4.76	1.12–14.22
	MMP2/TIMP2	>	>	>	<	>	<
Area of MMP9 in tunica media of aorta (%)	Mean ± SD	3.46 ± 2.74	6.57 ± 5.83	1.17 ± 0.7	7.16 ± 5.67	1.63 ± 1.57	20.79 ± 17.85
	Median	2.12 ^^^	3.18 ^C,∆,^^	1.17 ^T,M,^^	6.9 ^C,∆^	1.23 ^T,M,^^	18.94 *^,T,C,∆^
	Min–Max	0.56–11.8	0.81–18.4	0.10–2.64	0.81–18.92	0.21–5.5	2.07–66.2
Area of TIMP1 in tunica media of aorta (%)	Mean ± SD	2.18 ± 1.09	7.19 ± 4.4	6.71 ± 5.84	13.01 ± 9.08	1.53 ± 1.02	11.75 ± 9.48
	Median	2.18 ^T,M,^^	6.83 *^,∆^	3.71 ^∆^	11.72 *^,∆^	1.34 ^T,C,M,^^	11.03 *^,∆^
	Min–Max	0.53–4.54	0.41–3.42	2.16–0.98	2.23–0.28	0.16–4.39	0.72–31.58
	MMP9/TIMP1	=	<	<	<	=	>

AM: arithmetic mean; SD—standard deviation; *n*—number of measured areas; Min–Max—minimum and maximum values. * *p* < 0.05 vs control; ^T^ *p* < 0.05 vs. TRG; ^C^ *p* < 0.05 vs. CRG; ^M^ *p* < 0.05 vs. MRG; ^∆^ *p* < 0.05 vs. CMG; ^^^ *p* < 0.05 vs. TM.
